# Expression of anoctamin 7 (ANO7) is associated with poor prognosis and mucin 2 (MUC2) in colon adenocarcinoma: a study based on TCGA data

**DOI:** 10.5808/gi.23071

**Published:** 2023-12-29

**Authors:** Chen Chen, Siripat Aluksanasuwan, Keerakarn Somsuan

**Affiliations:** 1 Medical Science Graduate Program, Faculty of Medical Science, Naresuan University, Phitsanulok 65000, Thailand; 2 Cancer and Immunology Research Unit (CIRU), Mae Fah Luang University, Chiang Rai 57100, Thailand; 3 School of Medicine, Mae Fah Luang University, Chiang Rai 57100, Thailand

**Keywords:** anoctamin 7, colon adenocarcinoma, colorectal cancer, mucin 2, prognosis

## Abstract

Colon adenocarcinoma (COAD) is the predominant type of colorectal cancer. Early diagnosis and treatment can significantly improve the prognosis of COAD patients. Anoctamin 7 (ANO7), an anion channel protein, has been implicated in prostate cancer and other types of cancer. In this study, we analyzed the expression of ANO7 and its correlation with clinicopathological characteristics among COAD patients using the Gene Expression Profiling Interactive Analysis 2 (GEPIA2) and the University of Alabama at Birmingham CANcer (UALCAN) databases. The GEPIA2, Kaplan-Meier plotter, and the Survival Genie platform were employed for survival analysis. The co-expression network and potential function of ANO7 in COAD were analyzed using GeneFriends, the Database for Annotation, Visualization and Integrated Discovery (DAVID), GeneMANIA, and Pathway Studio. Our data analysis revealed a significant reduction in ANO7 expression levels within COAD tissues compared to normal tissues. Additionally, ANO7 expression was found to be associated with race and histological subtype. The COAD patients exhibiting low ANO7 expression had lower survival rates compared to those with high ANO7 expression. The genes correlated with ANO7 were significantly enriched in proteolysis and mucin type O-glycan biosynthesis pathway. Furthermore, ANO7 demonstrated a direct interaction and a positive co-expression correlation with mucin 2 (MUC2). In conclusion, our findings suggest that ANO7 might serve as a potential prognostic biomarker and potentially plays a role in proteolysis and mucin biosynthesis in the context of COAD.

## Introduction

Colorectal cancer (CRC) is among the leading causes of cancer-related death, in which colon adenocarcinoma (COAD) is the major type of CRC [1]. It ranks as the second most common cancer in women and the third most prevalent in men. Men have approximately 25% higher incidence and mortality rates for CRC compared to women [2]. The burden of CRC prevalence and mortality is rapidly increasing worldwide, particularly in developed countries. Predictions suggest that by 2030, the global incidence of CRC will rise by 60%, resulting in over 2.2 million new cases and 1.1 million fatalities [3]. Patients with CRC and distant metastasis often do not respond well to conventional treatment, leading to a poor 5-year survival rate of less than 10% [4]. Therefore, it is essential to identify accurate and reliable prognostic factors for early CRC diagnosis to improve patient survival rates.

Anoctamin 7 (ANO7) belongs to the anoctamin family of Ca^2+^-activated Cl^–^ channels. The anoctamin family has ten isoforms [5]. Some members of the anoctamin family, like ANO1, have been linked to the pathogenesis of CRC. ANO1 has been identified as a target of honokiol that inhibits the proliferation of CRC cells [6]. Similarly, Li and colleagues discovered that ANO9 downregulation plays a crucial role in the tumorigenesis and progression of CRC [7]. ANO7 has been shown to be downregulated in metastatic disease, and reduced protein expression is related to high-grade prostate cancer [8-10]. However, the question of whether ANO7 plays a role in the pathogenesis of CRC remains unanswered.

With the advancement of cancer research, the release of multiple omics datasets and user-friendly bioinformatic tools has significantly amplified our analytical capabilities. Notably, The Cancer Genome Atlas (TCGA) provides an extensive repository of genomic and clinical data encompassing various cancer types [11]. Therefore, in this study, we conducted a bioinformatic analysis based on the TCGA database to attain a more in-depth perspective on the prognostic and functional significance of ANO7 in the context of COAD. Gene Expression Profiling Interactive Analysis 2 (GEPIA2) and the University of Alabama at Birmingham CANcer data analysis Portal (UALCAN) were used to explore the expression of ANO7 and its association with clinicopathologic characteristics of COAD patients. Survival analysis was conducted using GEPIA2, Kaplan-Meier (KM) plotter, and Survival Genie web tools. The GeneFriends, the Database for Annotation, Visualization and Integrated Discovery (DAVID), GeneMANIA, and Pathway Studio were employed to explore the potential role of ANO7 in COAD.

## Methods

### Analysis of differential expression of *ANO7* gene in COAD and normal tissues

The *ANO7* gene expression level in COAD tissue was investigated in comparison to normal tissue using the TCGA-COAD dataset through both the GEPIA2 (http://gepia2.cancer-pku.cn/) [12] and UALCAN databases (http://ualcan.path.uab.edu) [13]. Within the GEPIA2 platform, the differential expression analysis of the *ANO7* gene was conducted across 275 COAD tissues and 41 normal tissues using a one-way ANOVA. In the UALCAN database, the statistical distinction in ANO7 expression was evaluated between 286 COAD tissues and 41 normal tissues using a Student’s t-test with unequal variance.

### Analysis of association between *ANO7* expression and clinicopathological characteristics of COAD patients

The UALCAN database was utilized to explore the association between ANO7 mRNA expression and clinicopathological characteristics among a total of 286 COAD patients. These characteristics include age, race, gender, cancer stage, histological subtype, and nodal metastasis status. A statistical comparison was performed using a Student’s t-test with unequal variance within the database.

### KM survival curve analysis

The KM survival curve analysis of COAD patients, based on ANO7 expression, was conducted using several web tools, including GEPIA2, KM plotter (https://kmplot.com/analysis/) [14], and Survival Genie (https://bbisr.shinyapps.winship.emory.edu/SurvivalGenie/) [15]. In GEPIA2, a division of patients (n = 267) into two groups was performed based on the median expression value, distinguishing between low and high expression. Similarly, KM plotter was employed to perform survival analysis on 304 COAD patients. The "Auto select best cut-off" option was utilized to divide the low and high expression groups. KM curves for patients' overall survival were presented, along with the log-rank p-value and hazard ratio (HR). Additionally, survival analysis was conducted on the Survival Genie platform with a cohort of 453 colon cancer patients. They were categorized into low and high ANO7 expression groups using the martingale residuals method [15].

### Co-expression network and functional enrichment analyses

In order to comprehensively explore the potential role of ANO7 in colon cancer, we collected the top 100 genes that displayed a positive correlation with ANO7 (based on the Pearson correlation coefficient, r) in the TCGA-COAD dataset from GEPIA2 for further analyses. A co-expression network of ANO7-correlated genes was built using GeneFriends (https://genefriends.org/) [16] with a Pearson correlation threshold of 0.7. The gene ontology (GO) and Kyoto Encyclopedia of Genes and Genomes (KEGG) pathway enrichment analyses were conducted using DAVID tool (https://david.ncifcrf.gov/) [17,18]. False discovery rate (FDR) below 0.05 were considered significant. The enrichment results were visualized using Hiplot (https://hiplot.org) web tool [19]. Additionally, molecular interaction network of ANO7-correlated genes mapped into significantly enriched GO biological processes and KEGG pathways was created using Pathway Studio software version 12.5 (Elsevier Inc., Rockville, MD, USA) [20].

### Analysis of association between ANO7 and mucins in COAD

An interaction network of ANO7 and mucin (MUC) genes (MUC1 to MUC24) [21] was constructed using GeneMANIA (https://genemania.org/) [22]. The correlation between ANO7 and MUC2 gene expressions was analyzed in the COAD-TGCA dataset using GEPIA2. Additionally, KM survival analysis of MUC2 in COAD patients was performed using GEPIA2.

## Results

### Differential expression of *ANO7* gene in COAD and normal tissues

**Fig. 1.**
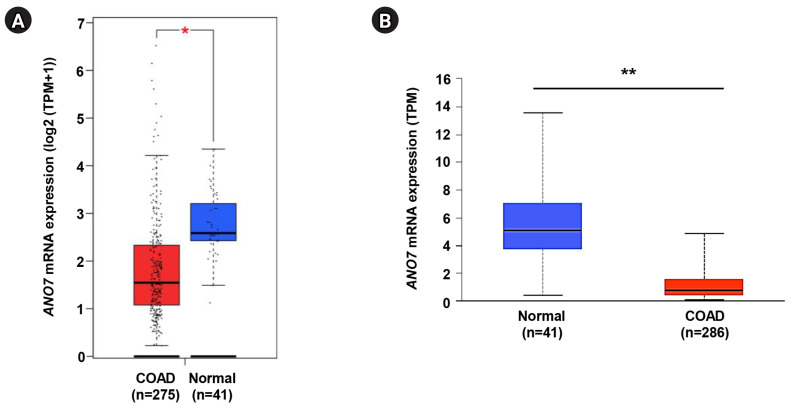
Differential expression of *ANO7* gene in COAD and normal tissues in GEPIA2 database (A) and UALCAN database (B). ANO7, anoctamin 7; COAD, colon adenocarcinoma; GEPIA2, Gene Expression Profiling Interactive Analysis 2; UALCAN, University of ALabama at Birmingham CANcer data analysis portal; TPM, transcripts per million. ^*^p < 0.05, ^**^p < 0.01.

The expression level of *ANO7* gene in COAD and normal tissues was explored in the GEPIA2 and UALCAN databases. The data consistently demonstrated a significant decrease in ANO7 mRNA expression in the COAD samples compared to the normal tissues across both databases (Fig. 1).

### Association between ANO7 expression and clinicopathological characteristics of COAD patients

**Fig. 2.**
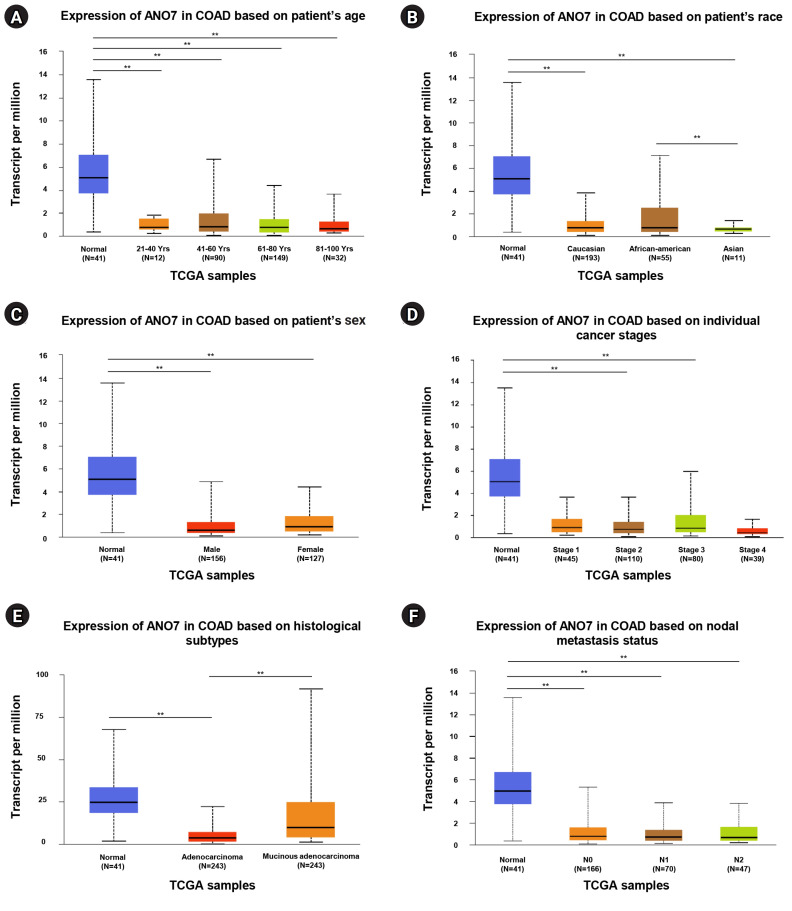
The ANO7 mRNA expression in COAD patients analyzed in UALCAN database based on patients’ age (A), race (B), sex (C), cancer stages (D), histological subtypes (E), and nodal metastasis status (F). ANO7, anoctamin 7; COAD, colon adenocarcinoma; TCGA, The Cancer Genome Atlas; UALCAN, University of ALabama at Birmingham CANcer data analysis portal. ^**^p < 0.01.

By utilizing UALCAN, we conducted an investigation into the association between ANO7 mRNA expression and various clinicopathological characteristics among COAD patients. The ANO7 expression demonstrated significant correlations with the patients’ race and histological subtypes. Specifically, ANO7 expression was notably higher in African-American patients compared to Asian patients (Fig. 2B). Furthermore, COAD patients diagnosed with mucinous adenocarcinoma exhibited significantly higher ANO7 expression levels compared to those with adenocarcinoma (Fig. 2E). No significant associations were observed between ANO7 expression and patient age (Fig. 2A), gender (Fig. 2C), cancer stages (Fig. 2D), or nodal metastasis status (Fig. 2E).

### Association between ANO7 expression and overall survival of COAD patients

**Fig. 3.**
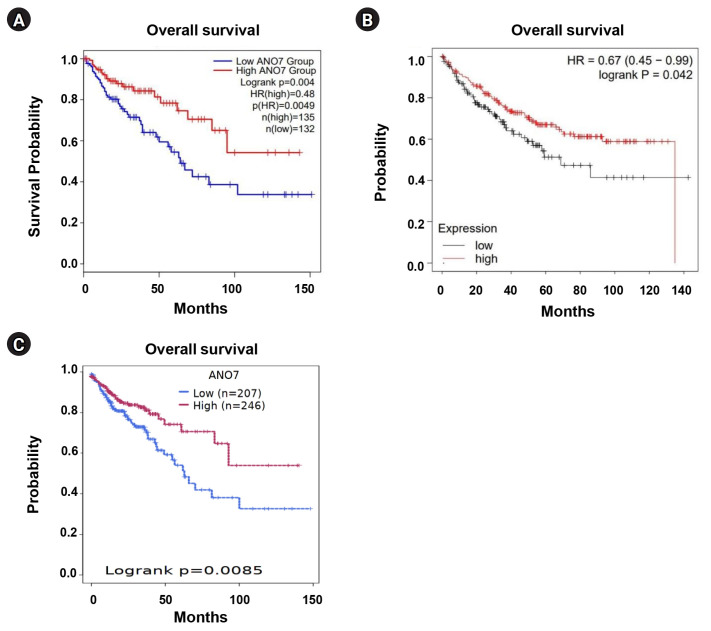
KM curves for overall survival in COAD patients with low and high ANO7 expression obtained from GEPIA2 (A), KM plotter (B), and the Survival Genie platform (C). ANO7, anoctamin 7; COAD, colon adenocarcinoma; GEPIA2, Gene Expression Profiling Interactive Analysis 2; HR, hazard ratio; KM, Kaplan-Meier.

We performed KM survival analysis for overall survival in COAD patients, categorized into low- and high-ANO7 expression groups, using GEPIA2, KM plotter, and Survival Genie web tools. Across these distinct web tools, the data consistently indicated that COAD patients with low ANO7 expression had significantly shorter overall survival compared to those with high ANO7 expression (Fig. 3).

### Co-expression network and functional enrichment of ANO7-correlated genes

**Fig. 4.**
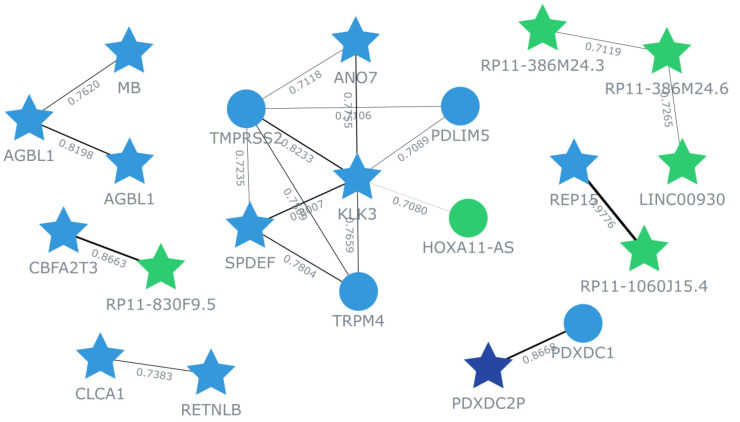
Co-expression network of ANO7-correlated genes in COAD created by GeneFriends. Each star symbolizes an input gene, while each circle represents an additional gene derived from the database. Line colors indicate the strength of correlation, accompanied by the Pearson correlation coefficient value. ANO7, anoctamin 7; COAD, colon adenocarcinoma.

**Fig. 5.**
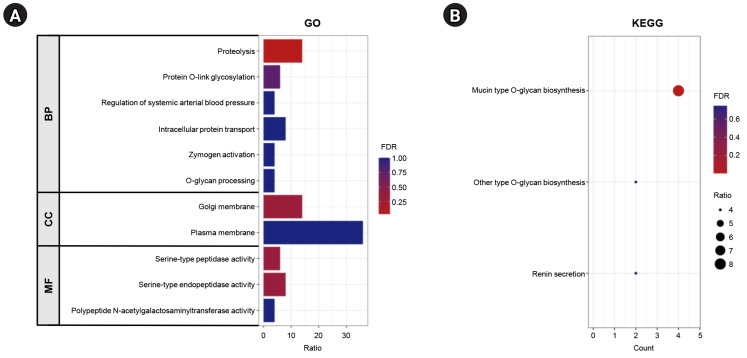
Functional enrichment analysis of ANO7-correlated genes in COAD analyzed by DAVID. (A) The GO enrichment terms of ANO7-correlated genes in COAD. (B) KEGG enrichment terms of ANO7-correlated genes in COAD. ANO7, anoctamin 7; COAD, colon adenocarcinoma; GO, gene ontology; BP, biological process; CC, cellular component; DAVID, The Database for Annotation, Visualization and Integrated Discovery; FDR, false discovery rate; KEGG, Kyoto Encyclopedia of Genes and Genomes; MF, molecular function.

**Fig. 6.**
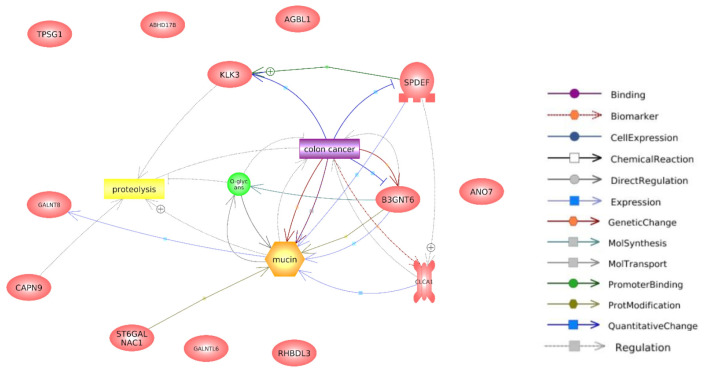
Molecular interaction network of ANO7-correlated genes with significant enrichment in proteolysis and mucin type O-glycan biosynthesis pathways created using Pathway Studio. ANO7, anoctamin 7.

The top 100 genes that exhibited a positive correlation with ANO7 in the TCGA-COAD dataset were selected for subsequent co-expression network and functional enrichment analyses. A list of these ANO7-correlated genes is summarized in Supplementary Table 1. The results obtained from GeneFriends showed that ANO7 displayed a strong co-expression correlation with kallikrein-related peptidase 3 (KLK3) (r = 0.78) and transmembrane serine protease 2 (TMPRSS2) (r = 0.71) (Fig. 4). Enrichment analysis data highlighted significant enrichments in the GO biological process term "proteolysis" (FDR = 0.038) and the KEGG pathway term "mucin type O-glycan biosynthesis" (FDR < 0.001) (Fig. 5, Supplementary Table 2). Further analysis using Pathway Studio elucidated a molecular and functional network of ANO7-correlated genes related to proteolysis and mucin dynamics in colon cancer (Fig. 6).

### Association between ANO7 and MUCs in COAD

**Fig. 7.**
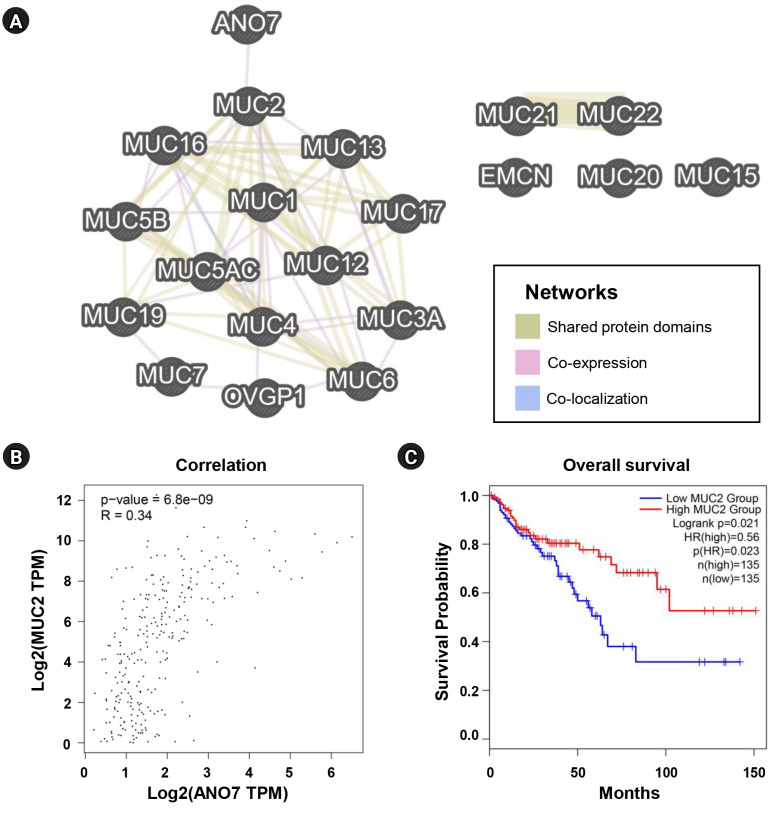
Association between ANO7 and MUCs in COAD. (A) Interaction network of ANO7 and all MUC genes (MUC1 to MUC24) constructed by GeneMANIA. (B) Correlation between ANO7 expression and MUC2 expression in GEPIA2. (C) KM curves for overall survival in COAD patients with low and high MUC2 expression obtained from GEPIA2. ANO7, anoctamin 7; COAD, colon adenocarcinoma; GEPIA2, Gene Expression Profiling Interactive Analysis 2; HR, hazard ratio; KM, Kaplan-Meier; MUC, mucin.

We further investigated the potential association between ANO7 and MUCs using GeneMANIA and GEPIA2 databases. Among all MUC genes, ANO7 showed a direct interaction only with MUC2, not with other MUCs (Fig. 7A). The result from GEPIA2 also indicated a significant correlation between ANO7 expression and MUC2 expression in the TCGA-COAD database (Fig. 7B). Following the same trend as ANO7, COAD patients with low MUC2 expression exhibited a notably shorter overall survival compared to those with high MUC2 expression (Fig. 7C). 

## Discussion

ANO7 is recognized as a prostate-specific protein. It has been proposed as both a diagnostic and therapeutic target for prostate cancer [8-10,23]. Furthermore, a growing body of evidence has recently demonstrated the pathogenic role of ANO7 in several other cancers, including breast cancer, thyroid cancer, and neuroblastoma [24-26]. As a result, ANO7 holds promise as an intriguing target for cancer biomarkers.

Our data analysis revealed that ANO7 expression is significantly lower in COAD tissues compared to normal tissues. This reduced ANO7 expression was associated with disease progression, and patients with a lower ANO7 expression level were linked to a poorer prognosis. This observation aligns with prior studies in prostate cancer [9,10], implying that ANO7 could potentially serve as a predictive marker for poor survival among COAD patients.

While ANO7 is well-known as an anion channel protein, recent reports have unveiled its involvement in diverse biological processes, including vesicle transport and cell-contact interactions [23]. However, the functional role of ANO7 in COAD remains largely unknown. In our study, co-expression network analysis showed that ANO7 had a strong co-expression correlation with the proteolytic enzymes KLK3 and TMPRSS2. Enrichment analysis also revealed a significant enrichment of ANO7-correlated genes within the proteolysis pathway. This is consistent with previous data suggesting that proteolysis contributes to extracellular matrix (ECM) degradation, invasion, and metastasis, as well as the malignant transformation of CRC [27]. Furthermore, KLKs are recognized as hallmarks of cancers [28]. KLK3, known as prostate-specific antigen (PSA), serves as a possible biomarker for various types of cancers [29-31]. Previous studies have showed that PSA is expressed in colon cancer tissues [32,33] Serum PSA level holds prognostic significance in women with CRC, as patients with low values of percent free PSA exhibited a poor survival outcome [34]. A recent study has identified ANO7 along with KLK3 in extracellular vesicles isolated from seminal plasma [35]. Taken together, these findings suggested a promising avenue for further investigating the cooperative roles of ANO7 and KLK3 in the proteolytic process and their potential as diagnostic markers in COAD.

According to KEGG pathway analysis, ANO7-correlated genes were significantly enriched in the mucin type O-glycan biosynthesis pathway. The mucin protein family consists of 24 members (MUC1 to MUC24) and plays important roles in various physiologic and pathogenic processes [21]. Aberrant expression and glycosylation of mucins are linked to CRC development and progression [36]. Among MUC family members, our interaction network analysis highlighted a potential relationship between ANO7 and MUC2. Their positive co-expression correlation was confirmed in the TCGA-COAD dataset. Low expressions of ANO7 and MUC2 were associated with a poor survival outcome for COAD patients. These findings align with previous studies that reported low MUC2 expression and its association with a poor prognosis in CRC [37-39]. In addition, a previous study has reported negative immunohistochemical expressions of MUC2 and KLK3 in primary signet-ring cell/histiocytoid carcinoma of the eyelid [40]. These data suggested the potential relationship among ANO7, MUC2, and KLK3. However, the role of ANO7 in the regulation of mucin biosynthesis and these novel connections need further elucidation.

Our present study has demonstrated that conducting bioinformatic analyses on publicly available omics datasets can yield valuable insights into the prognostic significance and potential function of ANO7 in COAD. However, it is important to note that our analysis primarily relied on the TCGA dataset. The inclusion of independent cohort studies in future investigations would greatly enhance the robustness of the prognostic significance attributed to ANO7 in COAD. Moreover, further experimental studies are necessary to validate the insights derived from bioinformatics and provide a mechanistic understanding of ANO7 in the context of COAD. Lastly, future research to examine ANO7 protein expression levels in COAD tissues compared to normal tissues would lead to a more comprehensive understanding of the role of ANO7 in COAD and also serve to validate the transcriptomic insights gained from this study.

In summary, ANO7 expression was significantly decreased in COAD tissues, and its low expression was associated with poor patient survival. ANO7 may play a role in the regulation of proteolysis and mucin biosynthesis. It may serve as a potential biomarker for COAD.

## Supplementary Materials

Supplementary Table. 1. Expression of Anoctamin 7 (ANO7) Is Associated with Poor Prognosis and Mucin 2 (MUC2) in Colon Adenocarcinoma: A Study Based on TCGA Data

Supplementary Table 2. Expression of Anoctamin 7 (ANO7) Is Associated with Poor Prognosis and Mucin 2 (MUC2) in Colon Adenocarcinoma: A Study Based on TCGA Data
