# Supplementary material for: Expression of anoctamin 7 (ANO7) is associated with poor prognosis and mucin 2 (MUC2) in colon adenocarcinoma: a study based on TCGA data
Source: Genomics Inform. 2023 Dec 29;21(4):e46. doi: 10.5808/gi.23071 (PMC10788358; doi:10.5808/gi.23071)
Supplement: Supplementary Table. 1. — Expression of Anoctamin 7 (ANO7) Is Associated with Poor Prognosis and Mucin 2 (MUC2) in Colon Adenocarcinoma: A Study Based on TCGA Data [file gi-23071-Supplementary-Table-1.pdf]

Supplementary Materials – Table S1

Expression of Anoctamin 7 (ANO7) Is Associated with Poor Prognosis and Mucin 2 (MUC2) in Colon Adenocarcinoma: A Study Based on TCGA Data

Chen Chen, Siripat Aluksanasuwan, and Keerakarn Somsuan

Correspondence to Keerakarn Somsuan; keerakarn.som@mfu.ac.th

Supplementary Table S1. List of the top 100 ANO7-correlated genes in TCGA-COAD dataset from GEPIA2.

No.	Gene symbol	Gene ID	Description	Pearson correlation coefficient	Category
1	*REP15*	ENSG00000174236.3	RAB15 effector protein	0.86	Protein coding
2	*B3GNT6*	ENSG00000198488.10	Acetylgalactosaminyl-O-glycosyl-glycoprotein beta-1,3-N-acetylglucosaminyltransferase	0.84	Protein coding
3	*LRRC26*	ENSG00000184709.7	Leucine rich repeat containing 26	0.82	Protein coding
4	*RP11-616M22.12*	ENSG00000277010.1	Novel transcript, antisense to TPSG1	0.81	RNA gene
5	*KLK3*	ENSG00000142515.14	Kallikrein-related peptidase 3	0.78	Protein coding
6	*RETNLB*	ENSG00000163515.6	Resistin like beta	0.77	Protein coding
7	*HMGB1P49*	ENSG00000230519.2	High mobility group box 1 pseudogene 49	0.77	Pseudogene
8	*CCDC60*	ENSG00000183273.6	Coiled-coil domain containing 60	0.76	Protein coding
9	*CAPN9*	ENSG00000135773.12	Calpain 9	0.75	Protein coding
10	*RP11-386M24.6*	ENSG00000260337.3	Novel transcript	0.74	RNA gene
11	*GAU1*	ENSG00000255474.1	GALNT8 antisense upstream 1	0.73	RNA gene
12	*AC011523.2*	ENSG00000267968.1	Novel transcript, antisense to KLK15	0.72	RNA gene
13	*RP11-386M24.3*	ENSG00000258676.4	Novel transcript	0.71	RNA gene
14	*STXBP5-AS1*	ENSG00000233452.6	STXBP5 antisense RNA 1	0.71	RNA gene
15	*GALNTL6*	ENSG00000174473.15	Polypeptide N-acetylgalactosaminyltransferase-like 6	0.7	Protein coding
16	*LRRIQ4*	ENSG00000188306.6	Leucine-rich repeats and IQ motif containing 4	0.7	Protein coding
17	*NPDC1*	ENSG00000107281.9	Neural proliferation, differentiation and control, 1	0.7	Protein coding
18	*MRPS35-DT*	ENSG00000256377.5	MRPS35 divergent transcript	0.7	RNA gene
19	*RP11-575A19.2*	ENSG00000238246.1	Novel transcript	0.7	RNA gene
20	*RP11-443G13.2*	ENSG00000263674.1	Novel transcript, antisense to RHBDL3	0.7	RNA gene
21	*TMEM210*	ENSG00000185863.7	Transmembrane protein 210	0.69	Protein coding
22	*RP11-99J16__A.2*	ENSG00000244137.1	Novel transcript, antisense to CAPN9	0.69	RNA gene
23	*CACNA2D2*	ENSG00000007402.11	Calcium channel, voltage-dependent, alpha 2/delta subunit 2	0.68	Protein coding
24	*GALNT8*	ENSG00000130035.6	Polypeptide N-acetylgalactosaminyltransferase 8	0.68	Protein coding
25	*HEPACAM2*	ENSG00000188175.9	HEPACAM family member 2	0.68	Protein coding
26	*LINC00930*	ENSG00000258647.5	Long intergenic non-protein coding RNA 930	0.68	RNA gene
27	*RP11-550A5.2*	ENSG00000254431.1	Novel transcript	0.68	RNA gene
28	*LINC01550*	ENSG00000246223.8	Long intergenic non-protein coding RNA 1550	0.68	RNA gene
29	*LINC01079*	ENSG00000229609.1	Long Intergenic Non-Protein Coding RNA 1079	0.68	RNA gene
30	*CTD-2337A12.1*	ENSG00000251314.2	Uncharacterized LOC101929710	0.68	RNA gene
31	*FCGBP*	ENSG00000275395.4	Fc fragment of IgG binding protein	0.67	Protein coding
32	*RP11-830F9.5*	ENSG00000259881.1	Novel transcript, antisense to CBFA2T3	0.67	RNA gene
33	*IFT140*	ENSG00000187535.13	Intraflagellar transport 140	0.66	Protein coding
34	*FAM174B*	ENSG00000185442.12	Family with sequence similarity 174, member B	0.66	Protein coding
35	*KIAA1324*	ENSG00000116299.16	Endosome/lysosome-associated apoptosis and autophagy regulator 1	0.66	Protein coding
36	*GP9*	ENSG00000169704.4	Glycoprotein IX	0.65	Protein coding
37	*RHBDL3*	ENSG00000141314.12	Rhomboid, veinlet-like 3	0.65	Protein coding

38	*RP11-1060J15.9*	ENSG00000276261.1	Novel transcript, antisense to a lncRNA	0.65	RNA gene
39	*CBFA2T3*	ENSG00000129993.14	Core-binding factor, runt domain, alpha subunit 2; translocated to, 3	0.64	Protein coding
40	*TPSG1*	ENSG00000116176.6	Tryptase gamma 1	0.64	Protein coding
41	*CLCA1*	ENSG00000016490.15	Chloride channel accessory 1	0.64	Protein coding
42	*SGSM3*	ENSG00000100359.20	Small G protein signaling modulator 3	0.64	Protein coding
43	*AGBL1*	ENSG00000166748.12	ATP/GTP binding protein-like 1	0.64	Protein coding
44	*RP11-244F12.3*	ENSG00000259498.1	TPM1 antisense RNA	0.64	RNA gene
45	*CTD-2547H18.1*	ENSG00000254988.1	Novel transcript	0.64	RNA gene
46	*LA16c-395F10.2*	ENSG00000260989.1	Uncharacterized LOC105371046	0.64	RNA gene
47	*MLPH*	ENSG00000115648.13	Melanophilin	0.63	Protein coding
48	*RASD1*	ENSG00000108551.4	RAS, dexamethasone-induced 1	0.63	Protein coding
49	*RAB26*	ENSG00000167964.12	RAB26, member RAS oncogene family	0.63	Protein coding
50	*CHMP1AP1*	ENSG00000226662.2	Charged multivesicular body protein 1A pseudogene 1	0.63	Pseudogene
51	*TBILA*	ENSG00000261488.1	TGF-beta induced lncRNA	0.63	RNA gene
52	*DNPEP-AS1*	ENSG00000229525.1	DNPEP antisense RNA 1	0.63	RNA gene
53	*RP11-507B12.2*	ENSG00000259616.2	Novel transcript	0.63	RNA gene
54	*ST6GALNAC1*	ENSG00000070526.14	ST6 (alpha-N-acetyl-neuraminyl-2,3-beta-galactosyl-1,3)-N-acetylgalactosaminide alpha-2,6-sialyltransferase 1	0.62	Protein coding
55	*AC096574.4*	ENSG00000225057.2	Novel transcript	0.62	RNA gene
56	*CYP1B1-AS1*	ENSG00000232973.11	CYP1B1 antisense RNA 1	0.62	RNA gene
57	*GPR20*	ENSG00000204882.3	G protein-coupled receptor 20	0.61	Protein coding
58	*CCNJL*	ENSG00000135083.14	Cyclin J-like	0.61	Protein coding
59	*MB*	ENSG00000198125.12	Myoglobin	0.61	Protein coding
60	*KLK1*	ENSG00000167748.10	Kallikrein 1	0.61	Protein coding
61	*TMEM61*	ENSG00000143001.4	Transmembrane protein 61	0.61	Protein coding
62	*TBC1D2*	ENSG00000095383.19	TBC1 domain family, member 2	0.61	Protein coding
63	*AC093642.1*	ENSG00000280119.1	Uncharacterized FLJ38379	0.61	RNA gene
64	*CMIP*	ENSG00000153815.16	c-Maf inducing protein	0.6	Protein coding
65	*SYTL1*	ENSG00000142765.17	Synaptotagmin-like 1	0.6	Protein coding
66	*LYSMD4*	ENSG00000183060.15	LysM, putative peptidoglycan-binding, domain containing 4	0.6	Protein coding
67	*CRACR2B*	ENSG00000177685.16	Calcium release activated channel regulator 2B	0.6	Protein coding
68	*MTUS2*	ENSG00000132938.18	Microtubule associated tumor suppressor candidate 2	0.59	Protein coding
69	*GPRIN3*	ENSG00000185477.4	GPRIN family member 3	0.59	Protein coding
70	*ATOH1*	ENSG00000172238.4	Atonal homolog 1	0.59	Protein coding
71	*ERN2*	ENSG00000134398.12	Endoplasmic reticulum to nucleus signaling 2	0.59	Protein coding
72	*RP11-134E15.1*	ENSG00000258619.2	HERV-FRD provirus ancestral Env polyprotein (ERVFRDE1) pseudogene	0.59	Pseudogene
73	*RP11-25K19.1*	ENSG00000167912.5	TOX divergent transcript	0.59	RNA gene
74	*RP11-1398P2.1*	ENSG00000244459.2	Novel transcript	0.59	RNA gene
75	*LINC00261*	ENSG00000259974.2	Long intergenic non-protein coding RNA 261	0.59	RNA gene
76	*RP11-747H7.3*	ENSG00000260711.2	Novel transcript, intronic to CATSPERB	0.59	RNA gene
77	*SPDEF*	ENSG00000124664.10	SAM pointed domain containing ETS transcription factor	0.58	Protein coding
78	*AGBL1*	ENSG00000273540.1	AGBL carboxypeptidase 1	0.58	Protein coding
79	*PDXDC2P*	ENSG00000196696.12	Pyridoxal-dependent decarboxylase domain containing 2, pseudogene	0.58	Protein coding
80	*SIDT1*	ENSG00000072858.10	SID1 transmembrane family, member 1	0.58	Protein coding
81	*PTGER2*	ENSG00000125384.6	Prostaglandin E receptor 2	0.58	Protein coding
82	*MED15P5*	ENSG00000236595.1	Mediator complex subunit 15 pseudogene 5	0.58	Pseudogene
83	*RP11-328N19.1*	ENSG00000251350.1	Long intergenic non-protein coding RNA 2475	0.58	RNA gene
84	*AP001626.2*	ENSG00000235023.1	Novel transcript	0.58	RNA gene
85	*RP11-830F9.6*	ENSG00000205018.2	Novel transcript, antisense to CBFA2T3	0.58	RNA gene
86	*ABHD17B*	ENSG00000107362.13	Abhydrolase domain containing 17B	0.57	Protein coding
87	*NEURL1*	ENSG00000107954.10	Neuralized E3 ubiquitin protein ligase 1	0.57	Protein coding
88	*HDHD1P1*	ENSG00000234620.1	Haloacid dehalogenase-like hydrolase domain containing 1 pseudogene 1	0.57	Protein coding

89	*COLCA1*	ENSG00000196167.9	Colorectal cancer associated 1	0.57	Protein coding
90	*SLITRK6*	ENSG00000184564.8	SLIT and NTRK-like family, member 6	0.57	Protein coding
91	*AC004062.2*	ENSG00000251312.2	Eukaryotic translation elongation factor 1 beta 2 (EEF1B2) pseudogene	0.57	Pseudogene
92	*CDY18P*	ENSG00000232205.1	Chromodomain protein, Y-linked 18 pseudogene	0.57	Pseudogene
93	*CTD-2299E8.1*	ENSG00000251585.1	Ash2 (Absent, small, or homeotic)-like (Drosophila) (ASH2L) pseudogene	0.57	Pseudogene
94	*RN7SL565P*	ENSG00000276562.1	RNA, 7SL, cytoplasmic 565, pseudogene	0.57	Pseudogene
95	*MTND2P17*	ENSG00000258239.1	MT-ND2 pseudogene 17	0.57	Pseudogene
96	*RN7SL722P*	ENSG00000273940.1	RNA, 7SL, cytoplasmic 722, pseudogene	0.57	Pseudogene
97	*AC016694.2*	ENSG00000224917.1	Testis specific protein, Y-linked 1 (TSPY1), pseudogene	0.57	Pseudogene
98	*RP11-794P6.1*	ENSG00000255428.1	Uncharacterized LOC100132078	0.57	RNA gene
99	*AC007948.1*	ENSG00000263863.1	Novel transcript	0.57	RNA gene
100	*RP11-860B13.1*	ENSG00000256747.1	Novel transcript	0.57	RNA gene
