# Supplementary material for: Expression of anoctamin 7 (ANO7) is associated with poor prognosis and mucin 2 (MUC2) in colon adenocarcinoma: a study based on TCGA data
Source: Genomics Inform. 2023 Dec 29;21(4):e46. doi: 10.5808/gi.23071 (PMC10788358; doi:10.5808/gi.23071)
Supplement: Supplementary Table 2. — Expression of Anoctamin 7 (ANO7) Is Associated with Poor Prognosis and Mucin 2 (MUC2) in Colon Adenocarcinoma: A Study Based on TCGA Data [file gi-23071-Supplementary-Table-2.pdf]

Supplementary Materials – Table S2

Expression of Anoctamin 7 (ANO7) Is Associated with Poor Prognosis and Mucin 2 (MUC2) in Colon Adenocarcinoma: A Study Based on TCGA Data

Chen Chen, Siripat Aluksanasuwan, and Keerakarn Somsuan

Correspondence to Keerakarn Somsuan; keerakarn.som@mfu.ac.th

Supplementary Table S2. The details of GO enrichment analysis and KEGG pathway analysis of ANO7-correlated genes in COAD using DAVID.

ID	Term	Count	%	Genes	Fold Enrichment	FDR
<u>**GO biological process**</u>						
**GO:0006508**	**Proteolysis**	**7**	**14**	***CAPN9, ABHD17B, AGBL1, TPSG1, CLCA1, KLK3, RHBDL3***	**7.85**	**0.038443**
GO:0006493	Protein O-linked glycosylation	3	6	*B3GNT6, GALNTL6, GALNT8*	21.20	0.77371
GO:0003073	Regulation of systemic arterial blood pressure	2	4	*KLK1, KLK3*	67.64	1
GO:0006886	Intracellular protein transport	4	8	*TBC1D2, MLPH, SGSM3, SYTL1*	5.79	1
GO:0031638	Zymogen activation	2	4	*KLK1, KLK3*	36.42	1
GO:0016266	O-glycan processing	2	4	*B3GNT6, GALNT8*	23.10	1
<u>**GO cellular component**</u>						
GO:0000139	Golgi membrane	7	14	*ST6GALNAC1, B3GNT6, RAB26, HEPACAM2, GALNTL6, CBFA2T3, GALNT8*	4.53	0.289054
GO:0005886	Plasma membrane	18	36	*NPDC1, LRRC26, TPSG1, PTGER2, SYTL1, CACNA2D2, GPR20, GPRIN3, GP9, TBC1D2, RASD1, ABHD17B, FAM174B, RAB26, NEURL1, SLITRK6, SIDT1, CLCA1*	1.45	1
<u>**GO molecular function**</u>						
GO:0008236	Serine-type peptidase activity	3	6	*ABHD17B, TPSG1, KLK3*	22.40	0.324097
GO:0004252	Serine-type endopeptidase activity	4	8	*KLK1, TPSG1, KLK3, RHBDL3*	9.37	0.324097
GO:0004653	Polypeptide N-acetylgalactosaminyltransferase activity	2	4	*GALNTL6, GALNT8*	44.06	1
<u>**KEGG pathway**</u>						
**hsa00512**	**Mucin type O-glycan biosynthesis**	**4**	**8**	***ST6GALNAC1, B3GNT6, GALNTL6, GALNT8***	**78.38**	**0.000296**
hsa00514	Other types of O-glycan biosynthesis	2	4	*GALNTL6, GALNT8*	30.02	0.746597
hsa04924	Renin secretion	2	4	*PTGER2, CLCA1*	20.44	0.746597
